# FGF9 Alleviates the Fatty Liver Phenotype by Regulating Hepatic Lipid Metabolism

**DOI:** 10.3389/fphar.2022.850128

**Published:** 2022-04-20

**Authors:** Fanrong Zhao, Lei Zhang, Menglin Zhang, Jincan Huang, Jun Zhang, Yongsheng Chang

**Affiliations:** ^1^ Key Laboratory of Immune Microenvironment and Disease (Ministry of Education), Tianjin Key of Cellular Homeostasis and Disease, Department of Physiology and Pathophysiology, Tianjin Medical University, Tianjin, China; ^2^ Department of Basic Medicine, School of Medicine, Shihezi University, Shihezi, China

**Keywords:** lipid synthesis, fatty acid oxidation, lipogenesis, fatty liver, FGF9

## Abstract

Although the fatty liver has been linked to numerous impairments of energy homeostasis, the molecular mechanism responsible for fatty liver development remains largely unknown. In the present study, we show that fibroblast growth factors 9 (FGF9) expression is increased in the liver of diet-induced obese (DIO), db/db, and ob/ob mice relative to their respective controls. The long-term knockdown of hepatic FGF9 expression mediated by adeno-associated virus expressing FGF9-specific short hairpin RNA (AAV-shFGF9) aggravated the fatty liver phenotype of DIO mice. Consistently, downregulation of FGF9 expression mediated by adenovirus expressing FGF9-specific shRNA (Ad-shFGF9) in the primary hepatocyte promoted the cellular lipid accumulation, suggesting that FGF9 exerts its effects in an autocrine manner. In contrast, adenoviruses expressing FGF9 (Ad-FGF9) mediated FGF9 overexpression in the liver of DIO mice alleviated hepatic steatosis and improved the insulin sensitivity and glucose intolerance. Moreover, the liver-specific FGF9 transgenic mice phenocopied the Ad-FGF9-infected mice. Mechanistically, FGF9 inhibited the expression of genes involved in lipogenesis and increased the expression of genes involved in fatty acid oxidation, thereby reducing cellular lipid accumulation. Thus, targeting FGF9 might be exploited to treat nonalcoholic fatty liver disease (NAFLD) and metabolic syndrome.

## Introduction

The liver is a central metabolic organ that regulates hepatic lipid metabolism, including lipogenesis, fatty acid oxidation, and lipoprotein uptake and secretion (Browning and Horton, 2004). The excessive accumulation of triglycerides (TGs) in hepatocytes is the hallmark of NAFLD. The spectrum of NAFLD ranges from simple fatty liver (hepatic steatosis) to nonalcoholic steatohepatitis (NASH), which may lead to liver fibrosis and cirrhosis, resulting in increased morbidity and mortality (Brunt, 2010). NAFLD is closely associated with insulin resistance, obesity, and other metabolic diseases (Browning and Horton, 2004). Although the fatty liver has been attributed to abnormal energy metabolism, the molecular mechanisms underlying fatty liver development remain largely unknown.

*De novo* lipogenesis is remarkably induced in NAFLD patients, contributing to the excessive TG accumulation in the liver. Moreover, hepatic lipogenesis, which is normally inhibited in the fasting state, is relatively high under fasting conditions and fails to further increase after feeding in NAFLD patients (Diraison et al., 2003; Donnelly et al., 2005). Carbohydrate response element-binding protein (ChREBP) is a basic helix-loop-helix leucine-zipper transcription factor, which is highly expressed in the liver and has been shown to regulate hepatic lipogenesis *via* activating its target genes, including fatty acid synthase (FAS), acetyl-CoA carboxylase 1 (ACC1), and stearoyl CoA desaturase 1 (SCD1) (Jois and Sleeman, 2017). The liver-specific knockout of ChREBP in ob/ob mice improved hepatic steatosis and insulin resistance (Dentin et al., 2006). In contrast, overexpression of ChREBP in the liver of mice resulted in worsening of hepatic steatosis (Benhamed et al., 2012). These studies clearly suggest that ChREBP is a key mediator of hepatic steatosis.

Peroxisome proliferator-activated receptor γ (PPARγ) belongs to the nuclear receptor superfamily, which is highly expressed in adipose and required for the differentiation of preadipocytes to mature adipocytes. PPARγ is typically expressed in the liver at only 20% of the levels found in adipose tissue (Tontonoz et al., 1994). However, PPARγ expression is markedly induced in the severe fatty liver. Hepatic PPARγ deficiency remarkably alleviates the fatty liver phenotype (Matsusue et al., 2003), clearly indicating that PPARγ is capable of activating the expression of genes involved in TG accumulation in hepatocytes and promoting the development of fatty liver. However, PPARα, another member of the PPAR subfamily, stimulates fatty acid oxidation and improves lipoprotein metabolism by regulating the expression of genes involved in peroxisomal and mitochondrial β-oxidation pathways, fatty acid uptake, and triglyceride catabolism. Mice lacking PPARα accumulate a copious amount of hepatic TG and become hypoketonemic and hypoglycemic during fasting (Leone et al., 1999). Fibrates, the synthetic PPARα agonists, have been used to treat dyslipidemia (Lefebvre et al., 2006).

The family of fibroblast growth factors (FGFs) regulates a plethora of developmental processes and physiological functions (Beenken and Mohammadi, 2009). In mammals, 18 members of the FGF family are divided into two categories, namely, endocrine and paracrine. The FGF19, FGF21, and FGF23 subfamilies have been shown to function in an endocrine manner, dependent on the presence of Klotho proteins in their target tissues, to regulate glucose, bile acid, vitamin D, and phosphate homeostasis, while other members of the FGF family are considered paracrine factors and are known for their roles in tissue patterning and organogenesis during embryogenesis (Degirolamo et al., 2016). Previous studies suggest that FGF9 participates in palate formation, sex determination, and lung development (Colvin et al., 2001; Yin et al., 2011; Iwata et al., 2012). Interestingly, recently two studies showed that FGF9 regulates browning of white adipocytes and is associated with human obesity (Sun et al., 2019; Shamsi et al., 2020). However, the physiological role of FGF9 in other metabolically active tissues remains unexplored. In the present study, we show that fasting-induced FGF9 in the liver regulates hepatic lipid metabolism in a cell-autonomous manner. Targeting FGF9 signaling might be exploited to treat NAFLD and other metabolic diseases.

## Results

### The Expression of FGF9 is Dysregulated in the Liver of Mice With Hepatic Steatosis

FGF21 is a peptide hormone, secreted predominantly from the liver, and its expression is increased in the fasting state (Badman et al., 2007; Inagaki et al., 2007). FGF21 has been shown to regulate the metabolism of lipids and glucose, and FGF21 agonism holds significant potential for treatment of NASH (Ritchie et al., 2020).

**FIGURE 1:**
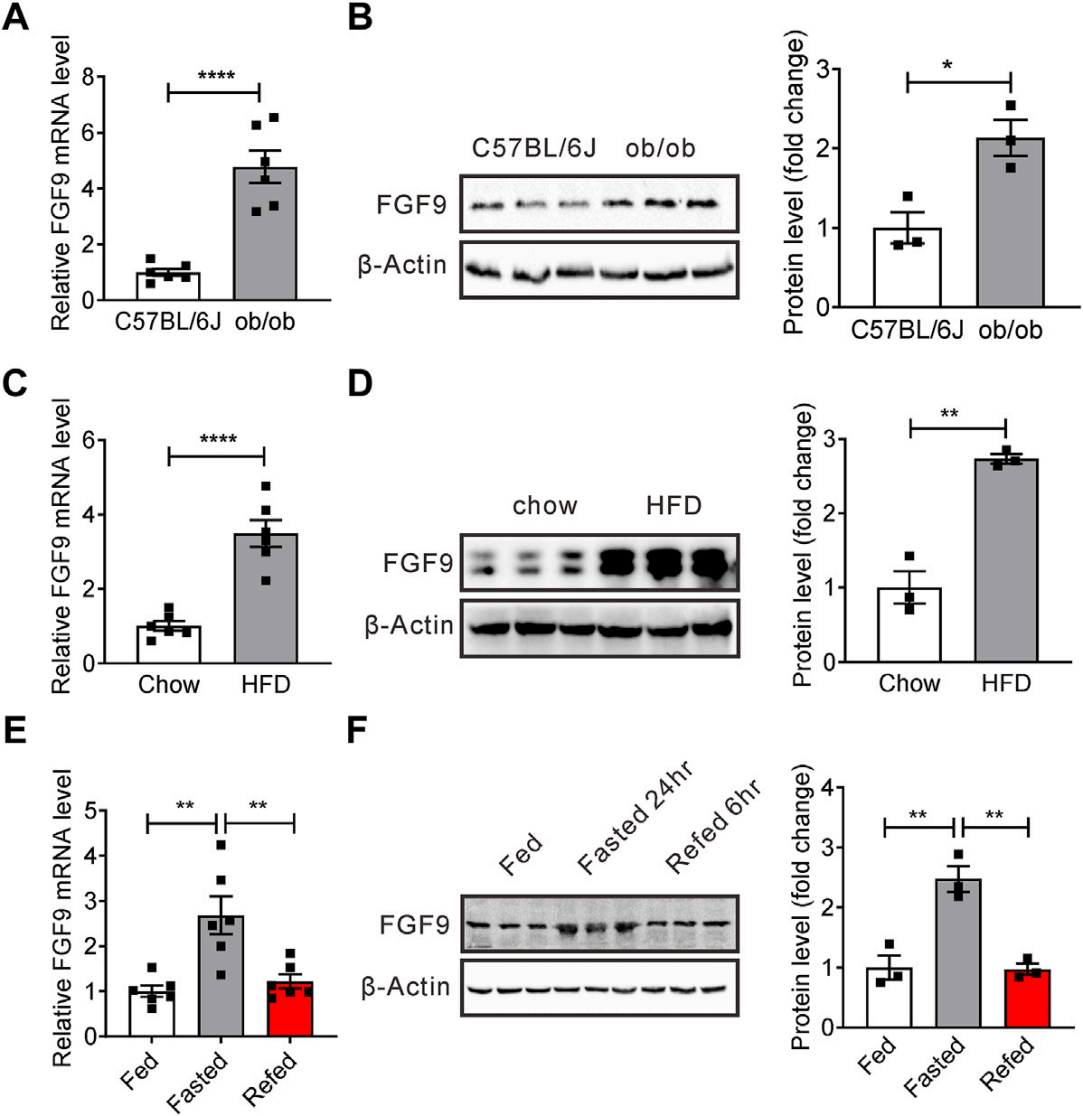
Expression of hepatic FGF9 is dysregulated in mice with fatty liver. **(A)** Quantitative PCR analysis of hepatic FGF9 in ob/ob mice (*n* = 6/group). **(B)** Representative Western blotting analysis of hepatic FGF9 in ob/ob mice. **(C)** Quantitative PCR analysis of hepatic FGF9 in C57BL/6J mice fed with a chow diet or high-fat diet for 3 months (DIO mice) (*n* = 6/group). **(D)** Representative Western blotting analysis of hepatic FGF9 in mice in **(C)**. **(E)** Quantitative PCR analysis of hepatic FGF9 in mice under *ad libitum*-fed, 24 hour-fasted, or 6 hour re-fed conditions (*n* = 6/group). **(F)** Representative Western blotting analysis of hepatic FGF9 in mice in **(E)**. All the data are presented as mean ± SEM, *p＜0.05, **p＜0.01, ****p < 0.0001, 2-tailed Student’s t-test **(A–D)**, 1-way ANOVA **(E,F)**.

To identify a novel secreted protein regulating systemic glucose and lipid metabolism, we have previously performed microarray analysis of livers of mice with hepatic steatosis (Shen et al., 2014). Preliminary data suggest that mRNA levels of FGF9, another member of FGF family, are also increased in the liver of ob/ob mice. Our real-time PCR and Western blotting data confirmed the microarray analysis (Figures 1A,B). We obtained similar results in DIO mice (Figures 1C,D). Interestingly, similar to FGF21, fasting-induced hepatic FGF9 expression, while refeeding, reversed this effect (Figures 1E,F).

These results indicate that FGF9 expression is regulated by different nutritional statuses, and FGF9 might have an important effect on hepatic glucose and lipid metabolism.

### Knockdown of FGF9 by AAV-shFGF9 in the Liver of DIO Mice Aggravated Hepatic Steatosis

**FIGURE 2:**
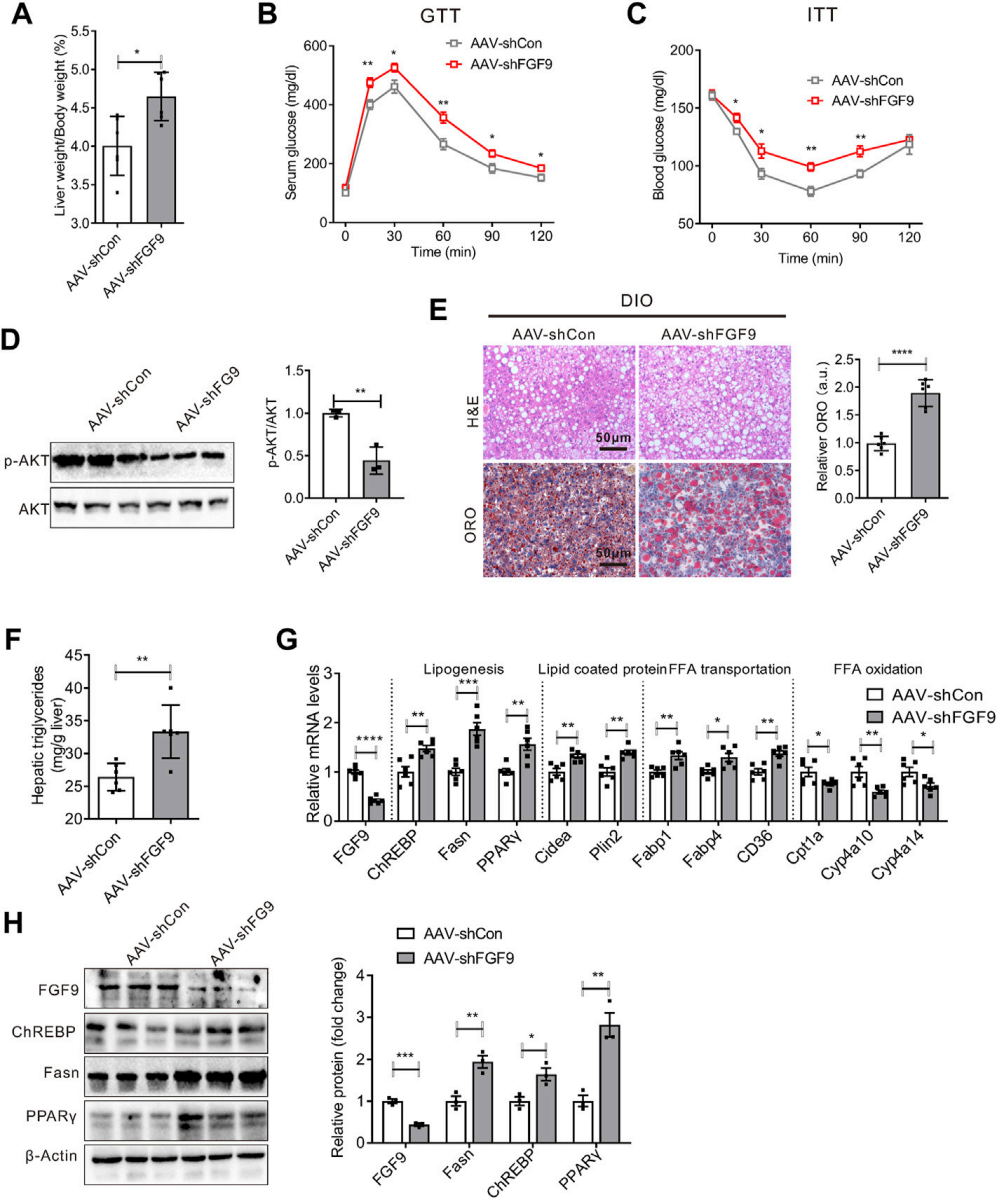
Knockdown of FGF9 in the liver of DIO mice exacerbated fatty liver phenotype. **(A)** Ratio of liver weight to body weight in C57BL/6J mice injected with AAV-shCon or AAV-shFGF9. AAV-infected mice were then fed with a high-fat diet (HFD) for 8 weeks (*n* = 6/group). **(B,C)** Blood glucose levels during GTTs **(B)** and ITTs **(C)** performed in mice in **(A)** (*n* = 6/group). **(D)** Representative Western blotting analysis of phosphorylated and total AKT in the liver of mice in **(A)** 20 min after intraperitoneal injection of insulin (0.75 U/kg). **(E)** Representative H&E (top panel) and Oil Red O staining (bottom panel) of livers from mice in **(A)**. **(F)** Hepatic TG levels in mice **(A)** (*n* = 6/group). **(G)**, Quantitative PCR analysis of genes involved in lipid metabolism in the liver of mice in **(A)** (*n* = 6/group). **(H)** Representative Western blotting analysis of genes involved in lipid synthesis in mice as described in **(A)**. All the data are presented as mean ± SEM, *p＜0.05, **p＜0.01, ***p＜0.001, ****p＜0.0001, 2-way ANOVA **(B,C)**, 2-tailed Student’s t-test **(A,D–H)**.

Given that FGF9 expression is induced in the liver of DIO mice, we explored the effects of long-term hepatic FGF9 deficiency on systemic metabolism. We first generated an adeno-associated virus expressing FGF9-specific short hairpin RNA (AAV-shFGF9) and infused AAV-shFGF9 into mice *via* the tail vein. Feeding a chow diet or high-fat diet after eight weeks, the mice were sacrificed for further studies. As a result, the knockdown of FGF9 did not affect hepatic lipid metabolism or systemic glucose metabolism in mice fed with a chow diet (Supplementary Figures S1B–E). However, an increased liver-to-body weight ratio was observed in mice injected with AAV-shFGF9 (Figure 2A). Moreover, AAV-shFGF9 treatment further aggravated glucose intolerance and insulin resistance induced by HFD (Figures 2B,C), and impaired hepatic insulin signaling (Figure 2D). Furthermore, H&E and Oil Red O staining revealed lipid accumulation in the liver (Figure 2E). The biochemical analysis also confirmed the significant increase in hepatic TG levels in AAV-shFGF9-injected mice (Figure 2F).

We also explored the molecular mechanism for the aggravated fatty liver phenotype in AAV-shFGF9-injected mice. We found that FGF9 knockdown in the liver increased the expression of genes involved in lipid synthesis (ChREBP, Fasn, and PPARγ) and fatty acid transport (Fabp1, Fabp4, and CD36). Meanwhile, AAV-shFGF9 treatment also decreased the expression of genes involved in lipid oxidation (Cpt1a, Cyp4a10, and Cyp4a14), as shown in Figures 2G,H. The results suggest that FGF9 plays an important role in the hepatic lipid metabolism.

### Knockdown of FGF9 Expression in Primary Hepatocyte Increased Cellular Triglyceride Contents

**FIGURE 3:**
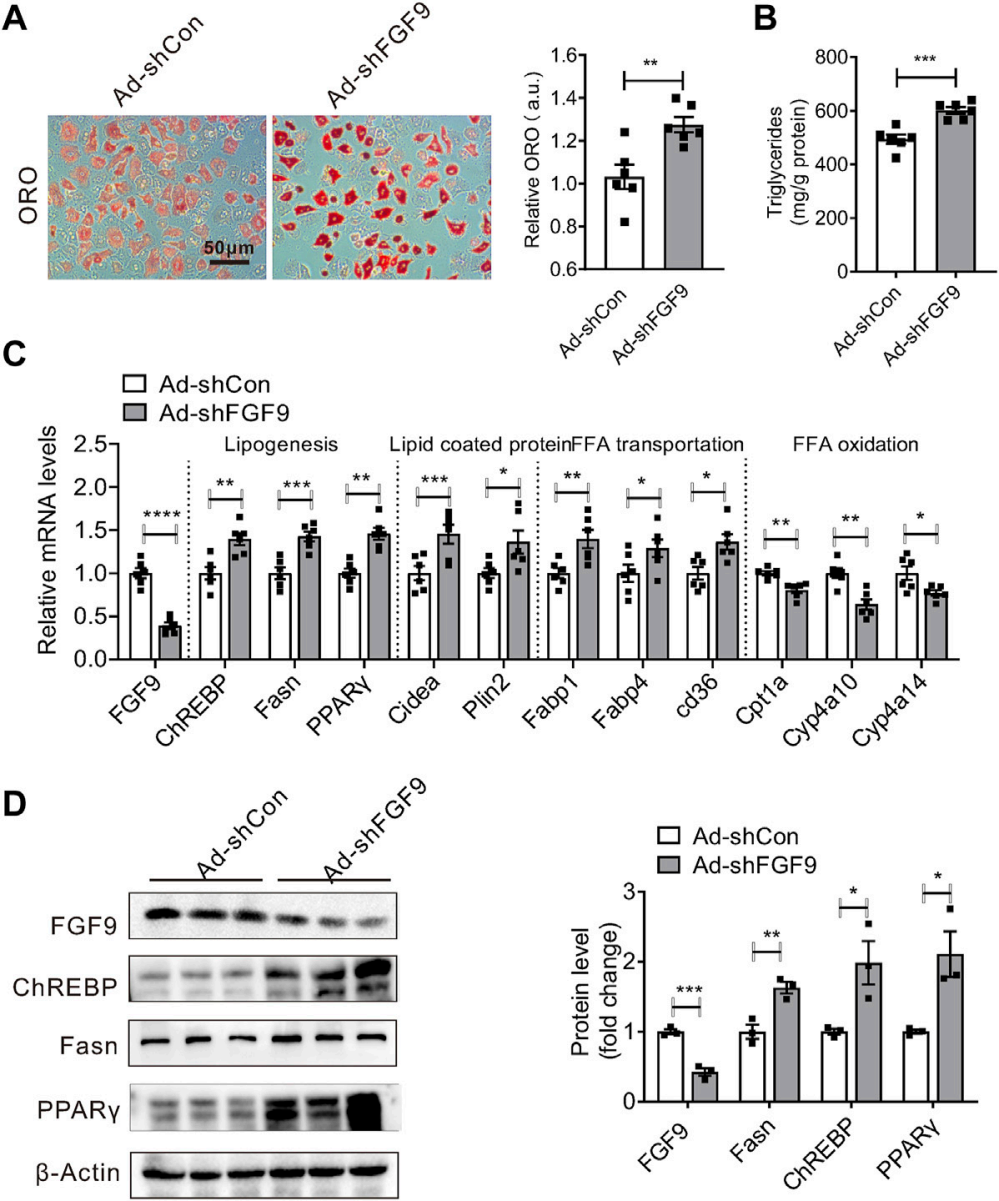
Knockdown of FGF9 in primary hepatocyte increased cellular TG content. **(A)** Representative Oil Red O staining of primary hepatocyte treatment with OA & PA for 24 h, followed by infecting with Ad-shCon or Ad-shFGF9 for 12 h prior to harvest for further analysis. **(B)** TG levels in primary hepatocyte in **(A)**. **(C)** Quantitative PCR analysis of genes involved in lipid metabolism in primary hepatocytes in **(A)**. **(D)** Representative Western blotting analysis of genes involved in lipid synthesis in primary hepatocytes in **(A)**. All the data are represented as mean ± SEM, *p < 0.05, **p＜0.01, ***p < 0.001, ****p＜0.0001, 2-tailed Student’s t-test **(A–D)**.

We next studied whether the effect of FGF9 deficiency on hepatic lipid metabolism is cell autonomous. We generated an adenovirus expressing FGF9-specific shRNA (Ad-shFGF9) and controlled adenovirus Ad-shCon. Primary hepatocytes treated with OA&PA, which stimulates cellular lipid synthesis, were infected with Ad-shFGF9 to observe the short-term effects of FGF9 deficiency on cellular lipid metabolism. As a result, the knockdown of FGF9 expression in primary hepatocytes increased lipid accumulation, as shown in Figures 3A,B.

Consistent with the aforementioned data obtained in DIO mice, we found that Ad-shFGF9 treatment increased the expression of several genes involved in lipogenesis such as ChREBP, Fasn, and PPARγ in primary hepatocytes. The expression of lipid-coated proteins such as cidea and plin2 was also increased. However, the expression of genes related to fatty acid oxidation such as Cpt1a, CYP4a10, and Cyp4a14 was down-regulated in primary hepatocytes infected with Ad-shFGF9. The Western blotting analysis confirmed real-time PCR results, as shown in Figures 3C,D. These results suggested that FGF9 regulating hepatic lipid metabolism is a cell-autonomous effect and FGF9 acts as an autocrine factor.

### Overexpression of FGF9 Mediated by Adenovirus in Primary Hepatocyte Reduced Cellular Lipid Contents

**FIGURE 4:**
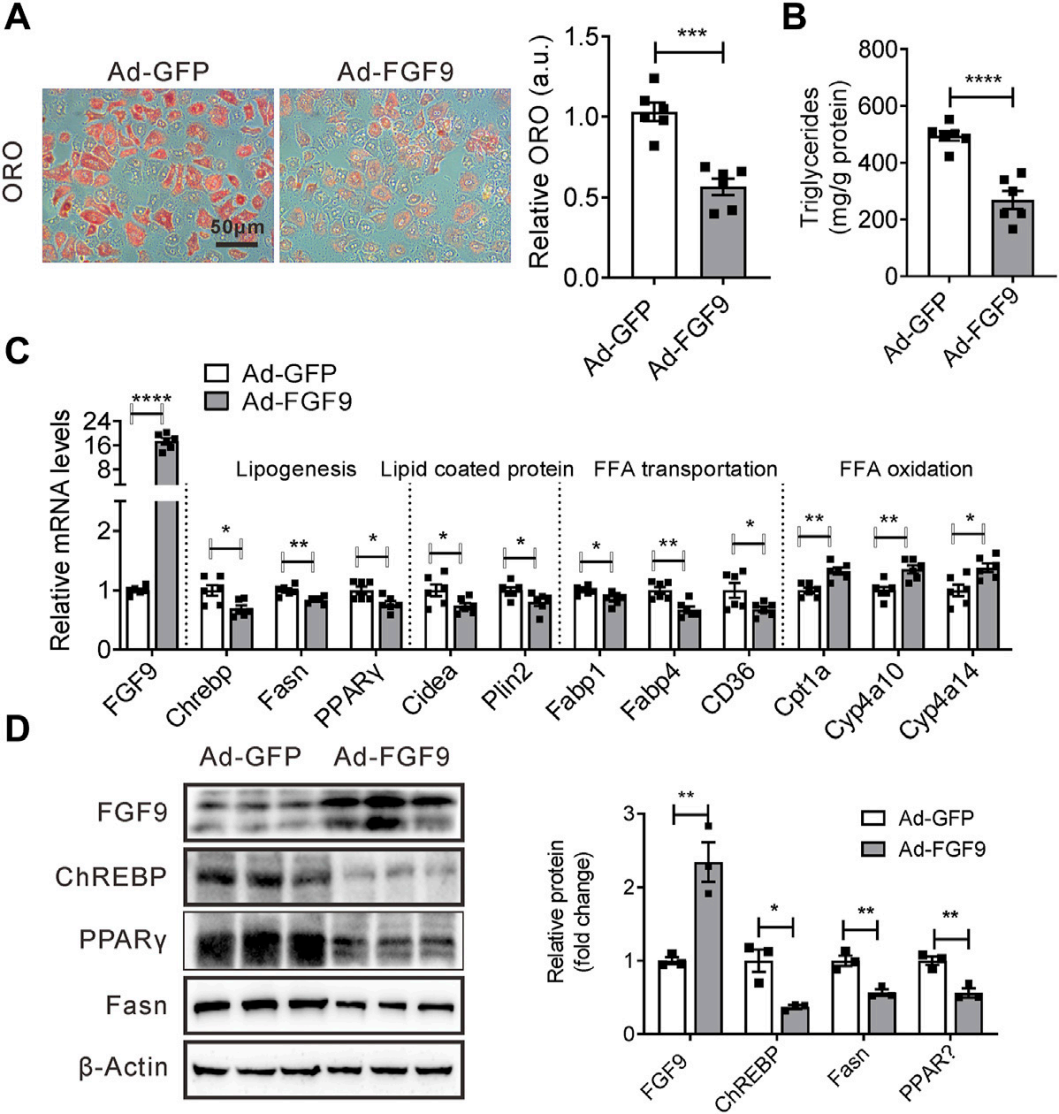
Overexpression of FGF9 in primary hepatocyte reduced cellular TG contents. **(A)** Representative Oil Red O staining of primary hepatocyte infected with Ad-GFP or Ad-FGF9 for 12 h, followed by treatment with OA & PA for 24 h prior to harvest for further analysis. **(B)** TG levels in primary hepatocyte in **(A)**. **(C)** Quantitative PCR analysis of genes involved in lipid metabolism in primary hepatocytes in **(A)**. **(D)** Representative Western blotting analysis of genes involved in lipid synthesis in primary hepatocytes in **(A)**. All the data are represented as mean ± SEM, *p < 0.05, **p < 0.01, ***p < 0.001, ****p＜0.0001, 2-tailed Student’s t-test **(A–D)**.

Given that FGF9 knockdown increased cellular Lipid accumulation, we next explored whether an increased expression of FGF9 in hepatocytes can reduce cellular lipid contents. To this end, the primary hepatocytes were infected with either Ad-FGF9 or Ad-GFP and followed by treatment with palmitic acid and oleic acid (OA&PA). As expected, cellular lipid accumulation induced by OA&PA was suppressed by Ad-FGF9 treatment (Figures 4A,B).

Correspondingly, Ad-FGF9 treatment decreased the expression of several genes involved in lipogenesis such as ChREBP, Fasn, and PPARγ, and fatty acid transport, including CD36, in primary hepatocytes, whereas it increased the expression of genes related to fatty acid oxidation such as Cpt1a, CYP4A10, and Cyp4a14 (Figure 4C). Meanwhile, the Western blotting analysis further confirmed these results (Figure 4D). The results suggested that the overexpression of FGF9 in primary hepatocyte reduced cellular lipid accumulation induced by OA&PA.

### Adenovirus-Mediated FGF9 Overexpression in the Liver of Diet-Induced Obese Mice Alleviates Hepatic Steatosis

To further explore the effect of FGF9 overexpression on hepatic lipid metabolism, we injected Ad-FGF9 or Ad-GFP (as a control) into chow or high-fat diet-fed C57BL/6J mice *via* the tail vein. As shown in Supplementary Figure S2, FGF9 overexpression in chow diet-fed mice did not significantly influence the systemic glucose metabolism or hepatic lipid metabolism.

**FIGURE 5:**
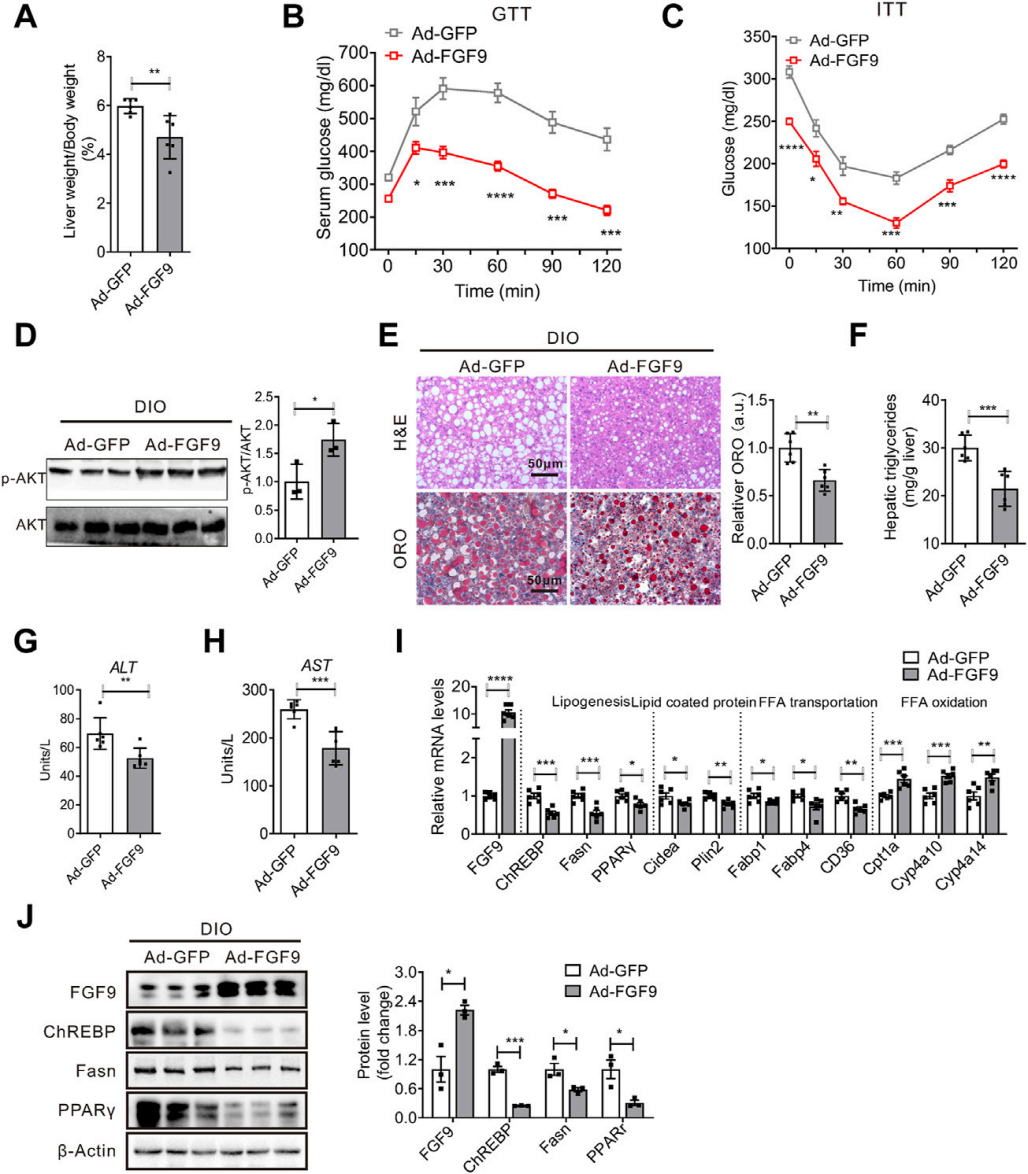
Adenovirus-mediated FGF9 overexpression in the liver of DIO mice alleviated NAFLD. **(A)** Ratio of liver weight to body weight in DIO mice infected with Ad-GFP or Ad-FGF9 for 15 days (*n* = 6/group). **(B,C)** Blood glucose levels during GTTs and ITTs performed in **(A)** (*n* = 6/group). **(D)** Representative Western blotting analysis of phosphorylated and total AKT in the liver of mice in **(A)** 20 min after intraperitoneal injection of insulin (0.75 U/kg). **(E)** Representative H&E staining (top panel) and Oil Red O staining (bottom panel) of liver sections from the mice in **(A)**. **(F)** Hepatic TG levels in mice in **(A)** (*n* = 6/group). **(G,H)** Plasma ALT and AST levels in mice in **(A)** (*n* = 6/group). **(I)** Quantitative PCR analysis of genes involved in lipid metabolism in the liver of mice in **(A)** (*n* = 6/group). **(J)** Representative Western blotting analysis of genes involved in lipid synthesis in mice in **(A)**. All the data are represented as mean ± SEM, *p < 0.05, **p < 0.01, ***p < 0.001, 2-way ANOVA **(B,C)**, 2-tailed Student’s t-test **(A,D–J)**.

However, the injection of Ad-FGF9 into DIO mice decreased the ratio of liver weight to body weight (Figure 5A). In contrast to the results obtained in AAV-shFGF9-infected DIO mice, Ad-FGF9 injection improves glucose intolerance as revealed by GTT experiments (Figure 5B), and enhanced insulin sensitivity, as revealed by ITT experiments (Figure 5C), and enhanced hepatic insulin signaling (Figure 5D). Notably, histological analysis of liver sections (H&E and Oil Red O staining) indicated that the fatty liver phenotype was markedly alleviated in DIO mice injected with Ad-FGF9 (Figure 5E). The biochemical analysis confirmed the reduced hepatic triglyceride content in Ad-FGF9-treated DIO mice (Figure 5F). At the same time, the decreased ALT and AST levels in DIO mice injected with Ad-FGF9 indicate that the overexpression of FGF9 alleviated the liver injury induced by HFD (Figures 5G,H).

Consistent with the results obtained *in vitro*, Western blotting and real-time PCR analysis confirmed that FGF9 overexpression in the liver of DIO mice inhibited the expression of genes involved in lipogenesis and increased the expression of genes related to fatty acid oxidation (Figures 5I,J). These results clearly suggest that FGF9 regulates the hepatic lipid metabolisms in DIO mice.

### Liver-Specific FGF9 Transgenic Mice Protected Against Hepatic Steatosis and Insulin Resistance Induced by HFD

**FIGURE 6:**
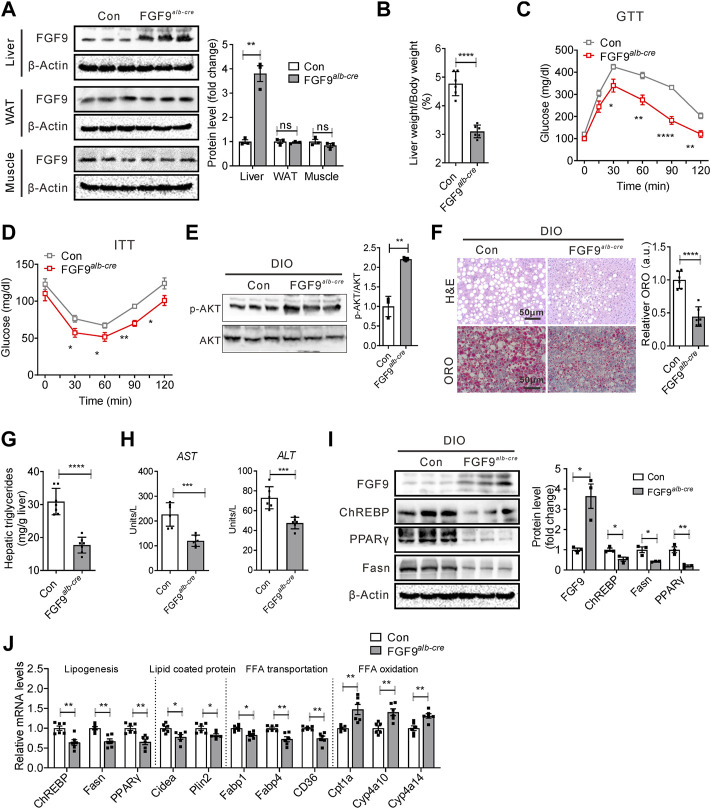
Liver-specific FGF9 transgene protected mice against NAFLD induced by HFD. **(A)** Representative Western blotting analysis of FGF9 protein levels in the liver, white adipose tissue (WAT), and muscle of FGF9 Rosa26 knockin mice (control) and liver-specific FGF9 transgenic mice (FGF9^*alb-cre*^). Data were normalized to β-actin. **(B)** Ratio of liver weight to body weight in control and FGF9^*alb-cre*^ mice fed with a HFD for 3 months (*n* = 6/group). **(C,D)** Blood glucose levels during GTTs and ITTs performed in mice in **(B)** (*n* = 6/group). **(E)** Representative Western blotting analysis of phosphorylated and total AKT in the liver of mice in **(B)** 20 min after intraperitoneal injection of insulin (0.75 U/kg). **(F)** Representative H&E staining and Oil Red O staining of livers from the mice in **(B)**. **(G)** Hepatic TG levels in mice in B (*n* = 6/group). **(H)** Plasma ALT and AST levels in mice in **(B)** (*n* = 6/group). **(I)** Representative Western blotting analysis of genes involved in lipid synthesis in the liver of mice in **(B)**. **(J)** Quantitative PCR analysis of genes involved in lipid metabolism in the liver of mice in B (*n* = 6/group). All the data are represented as mean ± SEM. *p < 0.05, **p < 0.01, ***p < 0.001, ****p＜0.0001, 2-way ANOVA **(C,D)**, 2-tailed Student’s t-test **(A,B,E–J)**.

To further confirm whether the overexpression of FGF9 inhibits NAFLD development induced by HFD, we generated liver-specific FGF9 transgenic mice (FGF9^*alb-cre*^) by crossing Alb-Cre mice and FGF9 Rosa26 knockin mice. Alb-Cre mice express the Cre-recombinant gene under the control of the albumin gene promoter. The FGF9 Rosa26 knockin mice were generated by the insertion of FGF9 cDNA downstream of the Rosa26 promoter and a loxP-stop-loxP cassette. The Western blotting analysis confirmed the FGF9 overexpression in the liver of FGF9^*alb-cre*^ (Figure 6A). FGF9 Rosa26 knockin mice were used as control mice. Again, when mice were fed a chow diet, FGF9 transgene in the liver did not affect markedly the systemic glucose metabolism and hepatic lipid metabolism (Supplementary Figure S3). However, when mice were fed a high-fat diet, we observed that the ratio of liver weight to body weight in FGF9^*alb-cre*^ mice was lower than that of control mice (Figure 6B). Moreover, FGF9 transgene enhanced the insulin sensitivity and improved glucose intolerance in diet-induced obese FGF9^*alb-cre*^ mice when compared to the control mice (Figures 6C–E). Furthermore, lipid accumulation in the liver was reduced from FGF9^*alb-cre*^ mice, as revealed by H&E and Oil Red O staining analysis (Figure 6F), and biochemical analysis (Figure 6G). Consistently, FGF9 transgene decreased the levels of serum ALT and AST, further confirming that the overexpression of FGF9 can alleviate the liver injury induced by HFD (Figure 6H).

We also examined the expression of genes involved in lipid metabolism in the liver of FGF9 transgenic mice. Western blotting and real-time PCR data further confirmed that FGF9 transgene inhibited the expression of lipogenic genes, while it enhanced the expression of genes related to fatty acid oxidation (Figures 6I,J). Collectively, these results clearly suggested that liver-specific FGF9 transgenic mice were resistant to HFD-induced NAFLD.

## Discussion

Given the rising incidence and high prevalence of NAFLD, the absence of approved therapies is striking. Currently, lifestyle changes, weight loss, and exercise are the main treatments available to modify the disease process. Thus, there is an urgent need of new drugs for treatment of NAFLD and NASH (Tiniakos et al., 2010; Rotman and Sanyal, 2017).

Previous studies indicated that FGF9 is involved in multiple developmental processes (Colvin et al., 2001; Yin et al., 2011; Iwata et al., 2012). Recently two studies showed that FGF9 regulates browning of white adipocytes and is associated with human obesity (Sun et al., 2019; Shamsi et al., 2020). Sun et al. (2019) reported that FGF9 expression in adipose tissue is reduced upon cold exposure and FGF9 treatment inhibited browning of white adipocytes. However, Shamsi et al. reported that cold exposure induces FGF9 expression in adipose tissues and FGF9 strongly induced UCP1 expression in adipocytes and preadipocytes, which is independent of adipogenesis and involves the FGFR3-PGE2-EP2/4 signaling pathway. Thus, it appears that FGF9 exerts its metabolic effects in an autocrine manner.

In the present study, we showed that hepatic FGF9 expression is upregulated in mice with the fatty liver, including HFD-induced obese and ob/ob mice relative to their respective control mice. Of note, we detected the two bands of FGF9 protein in livers of DIO mice, as revealed by Western blotting analysis, possibly due to the alternate splicing of FGF9 mRNA in DIO mice or different antibodies we used to detect an unspecific protein. Currently, two bands of FGF9 in nature remain unclear. Moreover, fasting also induces FGF9 expression in the mouse liver. Furthermore, FGF9 knockdown in the primary hepatocyte treated with OA and PA increased cellular lipid accumulation. Consistently, FGF9 knockdown in the liver of HFD-induced obese mice aggravated the symptoms of hepatic steatosis. These data suggest that FGF9 regulates hepatic lipid metabolism in an autocrine manner, as that observed in adipose tissues (Sun et al., 2019; Shamsi et al., 2020). Notably, FGF9 knockdown in the liver of chow diet-fed mice did not significantly change hepatic TG contents, indicating FGF9 exerts its effects in the context of high-fat stress. FGF9 expression is induced in the liver of both DIO mice and ob/ob mice. The overexpression of FGF9 in the liver of DIO mice improved insulin sensitivity and fatty liver phenotype. Whether FGF9 has same effects in ob/ob mice remains unexplored. Additionally, although our study shows that FGF9 regulates hepatic triglycerides contents in DIO mice, we did not characterize the lipid species influenced by FGF9. Ceramides are the best studied sphingolipids in relation to insulin resistance (Meikle and Summers, 2017). Thus, whether and how FGF9 influences ceramides contents deserve further study.

Multiple factors contribute to fatty liver development. Increased TG accumulation in the liver reflects an input/output imbalance of hepatic FFA metabolism. An increase in FFA delivery to the liver due to adipose tissue insulin resistance, and *de novo* lipogenesis driven by the hyperinsulinemia might cause fatty liver disease (Rotman and Sanyal, 2017). In contrast, the decrease in VLDL secretion or fatty acid β-oxidation also induces the fatty liver phenotype (Fabbrini et al., 2008; Zhang et al., 2013). In the present study, we found that FGF9 regulates hepatic lipid metabolism with several distinct mechanisms. FGF9 inhibits cellular lipid synthesis by repressing the expression of ChREBP, Fasn, and PPARγ, while it enhances fatty acid oxidation by inducing target genes of PPARα, including Cpt1a, Cyp4a11, and Cyp4a14. Of note, FGF9 did not influence the protein levels of PPARα (data not shown). We speculated that FGF9 might enhance PPARα transcriptional activity via posttranslational modification (Hinds et al., 2016) or increase the contents of its endogenous ligands. In addition, how FGF9 represses the expression of ChREBP and PPARγ remains unclear. Further studies are required to clarify this question.

FGF21, another member of FGF family, acting as an endocrine factor, is also induced directly by PPARα in the mouse liver in response to fasting and PPARα agonists. Induced FGF21, in turn, stimulates fatty acid oxidation and ketogenesis in the liver (Badman et al., 2007; Inagaki et al., 2007). Moreover, the adenoviral knockdown of FGF21 in the liver of mice fed a high-fat, low carbohydrate ketogenic diet (KD) caused the fatty liver and reduced serum ketones (Badman et al., 2007). Furthermore, injection of recombinant FGF21 proteins into DIO mice reversed hepatic steatosis due to FGF21 inhibition of hepatic lipogenesis (Xu et al., 2009). Our study indicates that FGF9 regulates hepatic lipid metabolism in an autocrine manner. Thus, FGF9 regulates hepatic lipid metabolism with similar mechanisms as FGF21. Fasting-induced FGF9 promotes hepatic fatty acid oxidation; meanwhile, it also inhibits hepatic lipogenesis, thereby improving the fatty liver phenotype.

NAFLD is the hepatic manifestation of the metabolic syndrome and becoming increasingly common with the rising incidence of obesity, diabetes, hyperlipidemia, and cardiovascular disease worldwide. Thus, the treatment of fatty liver should exhibit wider systemic effects and fit into a larger treatment of the syndrome (Ritchie et al., 2020). Our results suggest the beneficial effect of FGF9 on NAFLD, and targeting the FGF9 signaling pathway might be exploited to treat NAFLD or NASH. Of note, FGF9 has potential mitogenic activity (Tsai et al., 2002), which may lead to safety concerns. Fortunately, Shamsi et al. (2020) generated a modified FGF9 protein carrying K168Q/R173V/R177Q triple mutations in its HS-binding sites. This mutant reduced the molecule’s mitogenic potential without impacting its metabolic function. Thus, this FGF9 mutant might have potential for treatment of NAFLD.

## Materials and Methods

### Animal Treatment

Male C57BL/6J, ob/ob, db/db, and db/m mice at 8 weeks of age were purchased from the Model Animal Research Center of Nanjing University (Nanjing, China) and housed and maintained in 12 h light and dark photoperiods with a regular unrestricted diet.

For adenovirus infection, 8-week-old male C57BL/6J mice were fed a chow diet or HFD (D12492, Jiangsu Xietong Bio-engineering Co., Ltd., China) for 3 months, followed by injection with 1–2 × 10^9^ PFU per recombinant virus (Ad-GFP or Ad-FGF9) *via* the tail vein. Mice were fasted for 6 h and sacrificed for further analysis, 15 days after infection.

For AAV infection, mice were injected with 5×10^11^ vg of the AAV-shFGF9 or AAV-shCon via the tail vein, followed by feeding a chow diet or HFD for 8 weeks. Then, mice were sacrificed for further analysis.

FGF9 Rosa26 knockin mice were generated by the insertion of FGF9 cDNA downstream of the Rosa26 promoter and a loxP-stop-loxP cassette at Beijing Biocytogen Co., Ltd., and Alb–Cre mice (express the Cre-recombinant gene under the control of the albumin gene promoter) were provided by Hongbing Zhang (Institute of Basic Medical Sciences, Peking Union Medical College), and then liver-specific FGF9 transgenic mice were obtained by crossing Alb–Cre mice to FGF9 Rosa26 knockin mice. FGF9 Rosa26 knockin mice were used as controls. All the mice were fed on a chow diet or HFD for 3 months.

All the protocol related with animals was approved by the Animal Care and Use Committee of Tianjin Medical University (TMUaMEC) and conformed to criteria outlined in the National Institutes of Health (NIH; Bethesda, MD) Guide for the Care and Use of Laboratory Animals.

### Culture of Mouse Primary Hepatocyte and Adenoviral Infections

Briefly, 8-week-old male mice were anesthetized with bromethol and perfused with 0.5 mg/ml type II collagenase (Sigma-Aldrich) via the inferior vena cava to obtain hepatocytes as previously described (Wang et al., 2010). The cells were seeded at 6 well collagen-coated plates, and cultured with RPMI 1640 medium (H10394; Invitrogen) containing 10% (v/v) FBS (ExCell Bio, Shanghai, China), 50 units/ml penicillin and 50 μg/ml streptomycin (Penicillin–Streptomycin Solution, 100X, Solarbio Life Sciences, Beijing, China). After cell attachment for 4 h, a fresh medium was added after the unattached cells were washed away with PBS. For the adenovirus infection, primary hepatocytes were treated with 0.5 mM OA (CAS No. 112-80-1, Selleck) and 0.25 mM PA (CAS No. 57-10-3, Selleck) for 24 h before (Ad-shCon and Ad-shFGF9, MOI = 100) or after (Ad-GFP and Ad-FGF9, MOI = 100) adenovirus particle infection for 12 h.

### Triglyceride Content

Liver tissue of 100 mg was homogenized in 1 ml of 5% Nonidet P-40 dissolved in water, heated to 95°C for 5 min, centrifuged for 2 min at 13,000×g. A fluorometric assay kit was used to measure triglyceride levels according to the manufacturer’s guidelines (Applygen Technologies, Inc., Beijing, China). Finally, the levels of triglyceride levels were normalized to the weight of liver and expressed as mg of triglyceride/g of tissue weight.

### AAV-shRNA Viral Production and Purification

For the FGF9 knockdown studies, AAV gene delivery vectors were constructed by cloning FGF9 shRNA sequences into an AAV-shRNA-Ctrl plasmid (Addgene #85741, Watertown, MA), and AAV-shRNA-Ctrl encoding a nontargeting shRNA, was used as a control virus (AAV-shCon). For AAV-shFGF9, we generated the shRNA that would target FGF9 by using the Dharmacon siDESIGN center (http://www.dharmacon.com) as described (Yu et al., 2002), the antisense-loop-sense oligonucleotides synthesized, annealed, and subcloned into the Bbs1 and Xba1 sites of the AAV-shRNA-Ctrl plasmid. The AAV2/8 virus was generated by transfecting HEK-293T cells with pAAV2 insert containing either shRNA control or shRNA-FGF9 under the control of the mouse U6 promoter, pAAV2/8 (Addgene #112864, Watertown, MA) packaging plasmid expressing Rep/Cap genes, and pAdDeltaF6 (#112867).

The three plasmids were used for viral production with a triple-transfection, and the virus was purified according to the modified published methods (Challis et al., 2019). Briefly, triple plasmids were co-transfected to HEK-293T cells, and the cell culture medium was harvested at 72, 96, and 120 h. After purification by an iodixanol step gradient, the fraction containing the virus was desalted on 100 K concentrators with PBS as the diluent. AAV titers were determined by qPCR using primers targeting CMV. Each virus titer was calculated in genomes/ml with AAV-shRNA-Ctrl Luciferase at 2.4×10^13^ vg/ml, AAV-shFGF9 at 2.6×10^13^ vg/ml. The viruses were aliquoted into siliconized tubes and stored at −80°C.

The primers used for constructing AAV-shFGF9 are as follows:

shRNA FGF9 F: 5′-GAT​CCG​CAG​GAC​TGG​ATT​TCA​TTT​AGT​TCA​AGA​GAC​TAA​ATG​AAA​TCC​AGT​CCT​GCT​TTT​TTC​TCG​AGG-3’;

shRNA FGF9 R: 5′-AAT​TCC​TCG​AGA​AAA​AAG​CAG​GAC​TGG​ATT​TCA​TTT​AGT​CTC​TTG​AAC​TAA​ATG​AAA​TCC​AGT​CCT​GCG-3’.

### Recombinant Adenovirus Production and Purification

Adenoviruses expressing FGF9 was prepared as previously described (Luo et al., 2007). Briefly, the full-length mouse FGF9 gene was first cloned into the pAd-Track-CMV shuttle vector. The resultant plasmid was linearized by digesting with restriction endonuclease PmeI and subsequently transformed into competent cells, which were BJ5183 derivatives containing the adenoviral backbone plasmid pAdEasy-1. Recombinants were selected for kanamycin resistance, and recombination was confirmed by restriction endonuclease analyses. Overall, the confirmed recombinant adenovirus plasmids were digested with PacI to liberate both inverted terminal repeats and transfected into HEK-293A cells. Recombinant adenoviruses were typically generated within 14–20 days. Ad-shCon and Ad-shFGF9 were purchased from Hanbio Biotechnology Co., Ltd.

### Glucose Tolerance Tests and Insulin Tolerance Tests

For the GTTs, mice were injected with glucose (25% glucose, 1 g/kg) intraperitoneally after a 16-h fasting. For the ITTs, mice were fasted for 6 h and injected with insulin (0.75 U/kg; Novolinhuman insulin) intraperitoneally. Blood glucose levels were recorded using a glucose monitor (OneTouch; LifeScan, Inc., Milpitas, CA) at 0, 15, 30, 60, 90 and 120 min after glucose or insulin injection.

### Analysis of Hepatic Insulin Signaling

Mice were fasted for 16 h and intraperitoneally injected with insulin (0.75 U/kg). After twenty minutes, mice were euthanized, and the liver tissues were quickly excised, snap-frozen in liquid nitrogen, and stored at −80°C until use. For the evaluation of insulin signaling, primary antibodies against Ser-473-AKT (Cat No. AP0655, ABclonal) and total AKT (Cat No. A3145, ABclonal) were used for Western blotting analysis.

### Oil Red O Staining

The livers were mounted and frozen in Tissue-Tek O.C.T and sectioned at 5 μm. The frozen sections were air-dried, fixed in 10% neutral-buffered formalin, rinsed in tap water followed by 60% isopropanol, and stained in Oil Red O solution for 15 min. Then, the sections were further rinsed in 60% isopropanol, and the nuclei were stained with hematoxylin followed by aqueous mounting and cover slipping. For primary hepatocytes, the cells were fixed in 10% neutral-buffered formalin, rinsed in tap water followed by 60% isopropanol, and stained in the Oil Red O solution for 15 min.

All the sections were imaged using a color Axiocam105 camera with Zen 2 software attached to a Zeiss Axioplan microscope. The images were analyzed using ImageJ software.

### Hematoxylin-Eosin Staining

For H&E staining, liver tissues were fixed in 10% neutral-buffered formalin, embedded in paraffin, and cut into 5 μm sections. The sections were stained with hematoxylin and eosin followed by the manufacturer’s instructions (Polysciences, #24901). Briefly, the sections were deparaffinized and rehydrated in distilled water, followed by staining. Finally, the samples were dehydrated, cleared, and mounted, followed by imaging using light microscopy.

### Western Blotting Analysis

The homogenate of liver tissue or cells was prepared in lysis buffer (20 mM Tris–Cl pH7.5, 140 mM NaCl, 1 mM CaCl_2_ and MgCl_2_, 10 mM NaF, 1% NP-40, 10% glycerol, 2 mM Na-Vanadate, and 1 mM PMSF) supplemented with complete Protease Inhibitor Cocktail (cOmplete™, Sigma-Aldrich, Dallas, TX) for 30 min, centrifuged at 12,000 rpm at 4°C for 15 min. The protein samples were resolved by SDS-polyacrylamide gel electrophoresis and electrophoretically transferred to PVDF membranes. The membranes were blocked at room temperature for 2 h in 5% defatted milk dissolved in TBST (10 mM Tris–HCl, pH 7.4, and 150 mM NaCl), incubated at 4°C for overnight with the following antibodies: PPARγ (#AF6284, Affinity, 1:1000), FGF9 (#A6374, ABclonal, 1:500 and #ab206408, Abcam, 1:500), ChREBP (#A7630, ABclonal, 1:1000), Fasn (#DF6106, Affinity, 1:1000), or β-Actin (#AF7018, 1:10000), washed in TBST (0.1%Tween 20) for 15 min (repeated three times) and incubated with a goat anti-mouse (# S0002, Affinity) or goat anti-rabbit (# S0001, Affinity) IgG (H + L) HRP secondary antibody (1:5000 dilution in TBS) for 1 h at RT. Immunoreactivity was visualized and quantified by infrared scanning using the Odyssey system (LI-COR Biosciences).

### Quantitative PCR

Total RNA from either the mouse livers or the primary hepatocytes were extracted using the TRIzol-based method (Invitrogen). cDNA was prepared using the Applied Biosystems’ High-Capacity cDNA Reverse-Transcription Kit. Quantitative real-time reverse-transcriptase PCR (qRT-PCR) was performed using the SYBR Green I Q-PCR kit (TransGen) on a Bio-Rad IQ5 system. All gene expression data were normalized to 36B4 expression levels. All primer sequences are shown in Supplementary Table S1.

## Statistical Analysis

The quantitative data are represented as the mean ± SEM. A two-tailed, unpaired Student’s t-test was used for the pairwise comparison of genotypes or treatments. 1-way ANOVA and 2-way ANOVA were used when comparing 3 or more groups, as indicated in the figure legends. The graphs and analysis were performed using GraphPad Prism 7.0 software. *p* < 0.05 was considered statistically significant.

## Data Availability

The original contributions presented in the study are included in the article/Supplementary Material, further inquiries can be directed to the corresponding authors.

## Supplementary Material

Supplementary Table S1 List of specific primers used for real-time quantitative PCR gene expression analysis.
