# Supplementary material for: FGF9 Alleviates the Fatty Liver Phenotype by Regulating Hepatic Lipid Metabolism
Source: Front Pharmacol. 2022 Apr 20;13:850128. doi: 10.3389/fphar.2022.850128 (PMC9065278; doi:10.3389/fphar.2022.850128)
Supplement: Supplementary file 1 [file DataSheet2.docx]

ALL the source data for the information presented in tables and figures of manuscript can be obtained from the following links:

Figure 1 <https://www.jianguoyun.com/p/DZPWljYQg-mtChjhn7EE>

Figure 2 <https://www.jianguoyun.com/p/Df3w43cQg-mtChjln7EE>

Figure 3 <https://www.jianguoyun.com/p/DczNh8cQg-mtChjpn7EE>

Figure 4 <https://www.jianguoyun.com/p/DTdfoF4Qg-mtChjtn7EE>

Figure 5 <https://www.jianguoyun.com/p/DZMoUX8Qg-mtChjwn7EE>

Figure 6 <https://www.jianguoyun.com/p/DXnmhlEQg-mtChjyn7EE>

Figure S1 <https://www.jianguoyun.com/p/DYKyiD0Qg-mtChj0n7EE>

Figure S2 <https://www.jianguoyun.com/p/DbXZ0aYQg-mtChj2n7EE>

Figure S3 <https://www.jianguoyun.com/p/Da8tT-YQg-mtChj5n7EE>
