# Supplementary material for: FGF9 Alleviates the Fatty Liver Phenotype by Regulating Hepatic Lipid Metabolism
Source: Front Pharmacol. 2022 Apr 20;13:850128. doi: 10.3389/fphar.2022.850128 (PMC9065278; doi:10.3389/fphar.2022.850128)
Supplement: Supplementary file 2 [file DataSheet1.docx]

Supplementary Figures


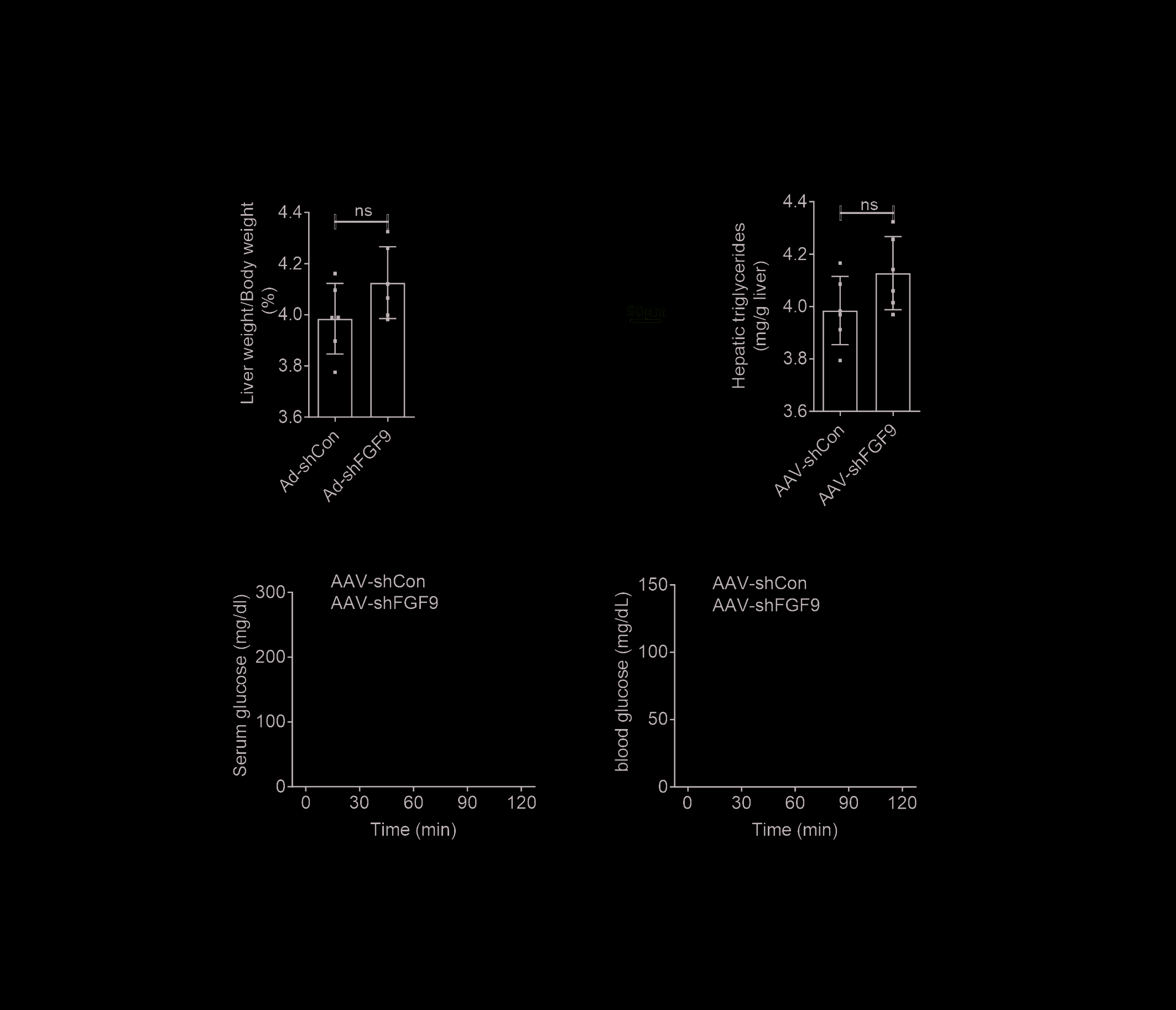


**Supplementary Figure 1.** **Knockdown of FGF9 in liver of C57 mice did not affect lipid metabolism when mice fed a chow diet.** **(A),** Quantitative analysis of hepatic FGF9 in mice injected with AAV-shCon or AAV-shFGF9. AAV-infected mice were then fed a chow diet for 8 weeks after injection (n=6/group)**.** **(B),** Representative H&E staining of liver sections from mice in **A**. **(C),** Hepatic TG levels in mice in **A** (n=6/group)**. (D, E)** Blood glucose levels during GTTs **(D)** and ITTs **(E)** performed in mice in **A** (n=6/group). All the data are presented as mean ± SEM, 2-way ANOVA **(D, E)**, 2-tailed Student's t-test **(A, C)**.


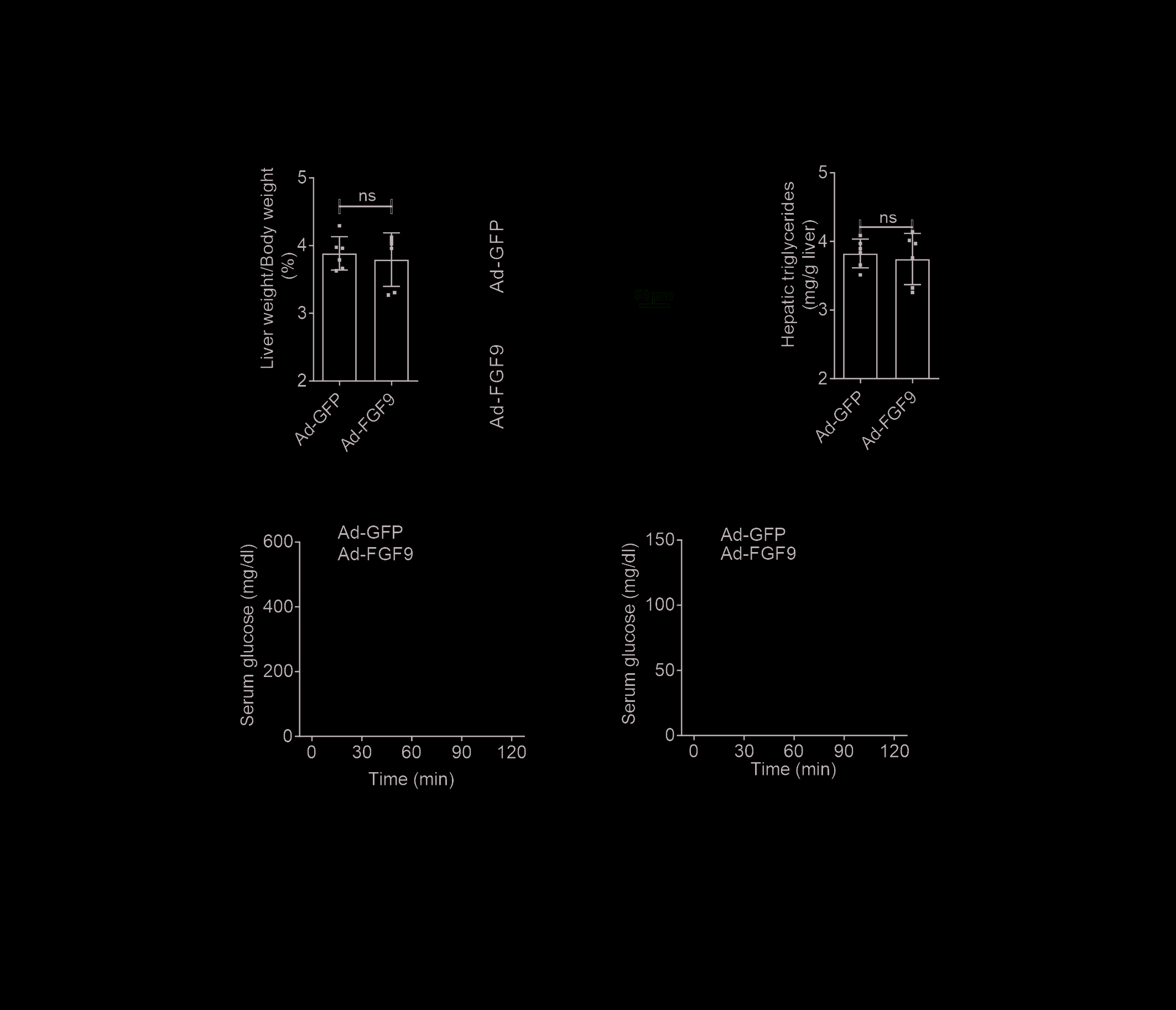


**Supplementary Figure 2. Adenovirus-mediated FGF9 overexpression in liver of C57 mice did not affect lipid metabolism when mice fed a chow diet. (A)** Ratio of liver weight to body weight in C57BL/6J mice infected with Ad-GFP or Ad-FGF9 for 15 days (n=6/group); **(B),** Representative H&E staining of liver sections from mice in **A**; **(C),** Hepatic TG levels in mice in **A** (n=6/group). **(D- E)** Blood glucose levels during GTTs **(D)** and ITTs **(E)** performed in mice in **A** (n=6/group). All the data are represented as mean ± SEM, 2-way ANOVA **(D, E)**, 2-tailed Student's t-test (**A, C**).


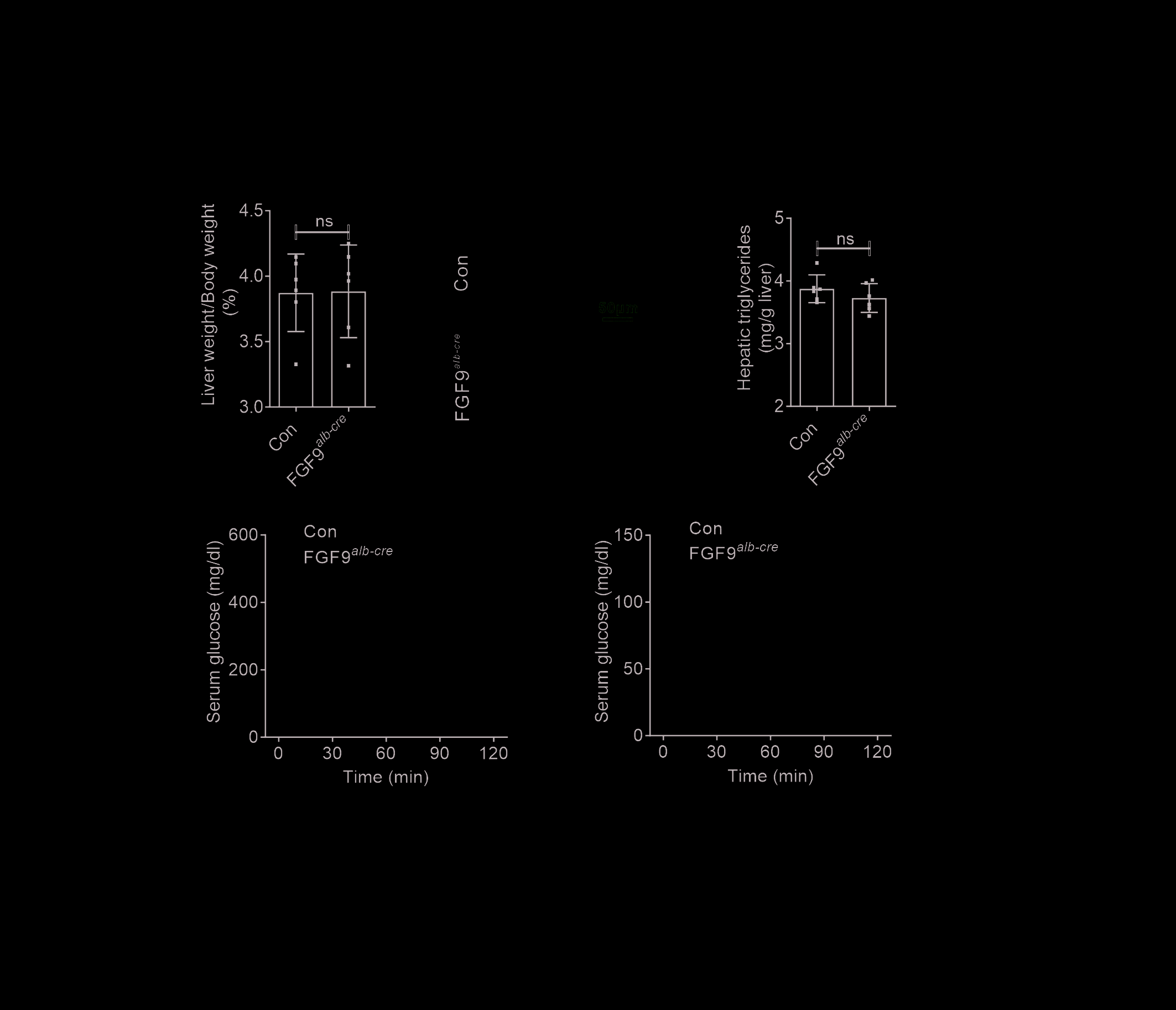


**Supplementary Figure 3. Liver-specific FGF9 transgene did not affect lipid metabolism when mice fed a chow diet. (A),** Ratio of liver weight to body weight in FGF9 Rosa26 knockin mice (control) and liver-specific FGF9 transgenic mice (FGF9*^alb-cre^*) fed a chow diet for 3 months (n=6/group); **(B),** Representative H&E staining of liver sections from mice in **A**. **(C),** Hepatic TG levels in mice in A (n=6/group). **(D-E),** Blood glucose levels during GTTs **(D)** and ITTs **(E)** performed in mice in **A** (n=6/group). All the data are represented as mean ± SEM, 2-way ANOVA **(D, E)**, 2-tailed Student's t-test (**A, C)**.
